# Optimization and validation of a consolidated Set of TaqMan qPCR assays for the surveillance of clinically relevant antibiotic resistance genes in environmental matrices

**DOI:** 10.1016/j.mex.2025.103600

**Published:** 2025-09-03

**Authors:** Sasikaladevi Rathinavelu, Karin Beck, Denise Lea Wälchli, Helmut Bürgmann

**Affiliations:** Eawag, Swiss Federal Institute of Aquatic Science and Technology, 6047 Kastanienbaum, Switzerland

**Keywords:** Environment, Antimicrobial resistance genes, Clinically relevant, qPCR, gBlocks, TaqMan assay, *aadA*, *aph(3″)-Ib*, *tetA*, *tetB*, *blaTEM*, *mecA*, *ermF*, *qnrS*, *mcr-1*, Surveillance, Detection, Quantification, Wastewater, Metagenome

## Abstract

The continued rise of antibiotic resistance and adoption of the One Health approach necessitates reliable methods for detection and quantification of antibiotic resistance genes (ARGs) in complex environmental matrices. Here we present a consolidated set of TaqMan quantitative PCR assays for quantification of clinically relevant and emerging ARGs in complex environmental matrices.

• We systematically designed five new primer sets, six TaqMan probes and verified and adapted four previously published relevant primer/probe sets from literature and evaluated their specificity *in silico* against current database.
• For external quantification, two sets of gBlock standard libraries were designed. We experimentally validated the specificity, sensitivity, and efficiency of the assays with positive strain control DNA, negative strain control DNA, general no target controls, extraction blank controls, negative controls, and environmental test samples (i.e., metagenomic DNA from complex environmental matrices) to comprehensively assess each assays’ performance.
• Optimization included iterative testing of both primer and probe concentration, annealing temperature, and annealing time. Results demonstrated robust and reliable detection and quantification of ARGs in clinical isolates and wastewater effluents with high sensitivity, specificity, and efficiency.

This makes the assays suitable for surveillance in wastewater or various environmental matrices, in support of efforts to mitigate dissemination of antibiotic resistance*.*

## Graphical abstract

**Figure:**
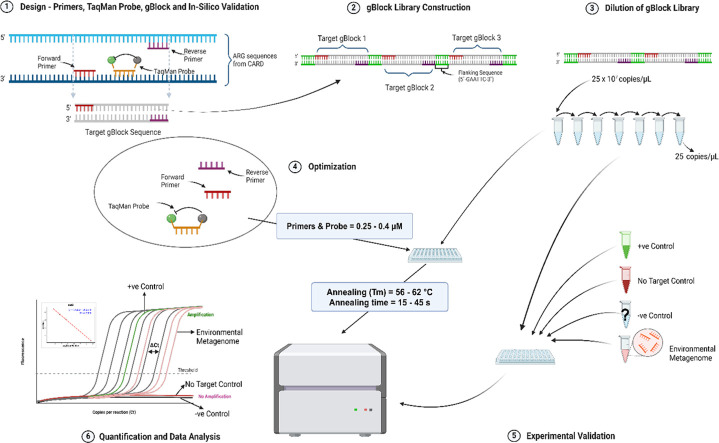

## Specifications table

**Table:** 

**Subject area**	*Environmental Science*
**More specific subject area**	Environmental Antimicrobial Resistance
**Name of your method**	qPCR Detection and Quantification of Clinically relevant Environmental Antibiotic Resistance Genes using TaqMan probes and gBlock standards*.*
**Name and reference of original method**	[1] P.M. Holland, R.D. Abramson, R. Watson, D.H. Gelfand, Detection of specific polymerase chain reaction product by utilizing the 5′—-3′ exonuclease activity of Thermus aquaticus DNA polymerase., Proceedings of the National Academy of Sciences. 88 (1991) 7276–7280. https://doi.org/10.1073/pnas.88.16.7276. [2] J. Conte, M.J. Potoczniak, S.S. Tobe, Using synthetic oligonucleotides as standards in probe-based qPCR, Biotechniques. 64 (2018) 177–179. https://doi.org/10.2144/btn-2018-2000.
**Resource availability**	Reagents: PolyA (0.5 mg/mL, Merck, Switzerland) Buffer AE (Elution buffer, Merck, Switzerland) Nuclease-free molecular grade water (Sigma-Aldrich, UK) LightCycler® 480 Probes Master (2x Conc.), LightCycler® 480 Probes Master H_2_O (Roche, Germany) gBlocks, primers, probes (Integrated DNA Technologies, Belgium) Filter: 0.22 µm, 47 mm cellulose ester S-Pak filter (Merck, Germany) DNA extraction kits: QIAmp® DNA Mini kit (Qiagen, Germany) DNeasy Power Water Kit (Qiagen, Germany) DNA Quantification: Qubit™ dsDNA BR Assay Kit (Thermo Fisher Scientific, USA) Analytical instruments: LightCycler®480 Roche (Roche, Switzerland) NanoDrop One Spectrophotometer (Thermo Fischer Scientific, USA) Qubit™ 4 Fluorometer (Thermo Fisher Scientific, USA) Consumables: Nuclease-free PCR tubes (Nippon Genetics, Europe) µltraAmp PCR Plates (384-Well 480 Plate, Sorenson™ Bioscience, USA) Software: LightCycler®480 Roche software (Version 1.5.1.62 SP3) Geneious prime (Version 2023.2.1) R statistical software (Version 4.3.2)

## Background

Antimicrobial resistance (AMR) is a critical global health threat, undermining our ability to treat infectious diseases and increasing the risk of severe illness and death. The emergence and spread of resistant pathogens are exacerbated by antibiotics overuse and misuse in human medicine, agriculture, and aquaculture [3]. The One Health approach recognizes the interconnectedness of human, animal, and environmental health, which is essential to tackling the AMR crisis. Environmental surveillance is vital for monitoring antibiotic-resistant bacteria (ARB) and genes (ARGs), which have become increasingly prevalent across various environmental matrices [4].

Accurate detection and quantification are key to understanding ARG spread, and distribution to devise mitigation strategies. Traditional methods for detecting ARB and ARGs in environmental matrices, like culture-based techniques [5] and conventional PCR with gel electrophoresis lack speed, sensitivity, and precision. Array PCR platforms (also available as commercial services) [6] allow high-throughput screening of a broad spectrum of ARGs but are limited by cost and complexity. Sequencing offers comprehensive resistome insights [6] and the potential to discover novel ARGs, but remains expensive, time-consuming, and less reliable for quantifying low-abundance ARGs. SYBR green qPCR is a widely used, straightforward, and cost-effective method for monitoring and quantification with a relatively short turnaround time. SYBR green-based assays cannot differentiate a specific target (e.g., ARG of interest) from PCR artifacts (e.g., primer dimers) as the dye binds to any double-stranded DNA [7], increasing the risk of false-positive results especially when applied to environmental DNA with diverse microbial communities and gene targets. To address these limitations, we developed a comprehensive set of TaqMan qPCR assays targeting suitable ARGs that combine clinical relevance and usefulness as environmental monitoring targets. The probe based TaqMan assay generates fluorescence signals only upon specific target amplification, significantly reducing non-specific amplification and false positives for more accurate and reliable ARG quantification [7]. TaqMan qPCR offers a cost-effective, rapid, precise, and targeted approach ideal for routine environmental AMR monitoring and quantification, enabling identification of hotspots or contamination events, informing targeted interventions, and supporting public health and environmental sustainability.

We adapted TaqMan probe chemistry to detect and quantify emerging ARGs, including *mcr-1, mecA, aadA, aph(3″)-Ib, tetA, tetB, ermF, blaTEM* and *qnrS.* These ARGs fall under WHO’s antibiotic categories: access *(aadA, aph(3″)-Ib, tetA, tetB, blaTEM),* watch *(mecA, ermF, qnrS)* and reserve *(mcr-1)* [8].

We used gBlock synthetic standards, which offer enhanced control over sequence composition, eliminate vector backbone sequences, and ensure consistency and reliability compared to plasmid standards [2,9]. These standards enable reproducibility and precise ARG quantification across assays, facilitating comparisons between experimental conditions. Primers and probes were validated with genomic DNA from clinical strains carrying the target ARGs, confirming their accuracy and reliability, and metagenomes from two wastewater plants to demonstrate the sensitivity, robustness, and reliability of the assays.

Our method addresses the need for a consistent, reliable, and rapid measurements of relevant ARGs in environmental matrices by designing novel primers/probes and optimizing existing ones. This approach improves sensitivity, specificity, and quantitative accuracy in AMR surveillance, supporting policy and regulatory decisions to mitigate AMR spread under the One Health approach.

## Method details

### Criteria for ARG selection

The ARGs *tetA, tetB, ermF, blaTEM, mecA, mcr-1, aadA, aph(3′')-Ib,* and *qnrS,* were chosen based on mobility, prevalence, WHO AWaRe classification, and environmental significance. *tetA* and *tetB* are common tet gene variants conferring tetracycline resistance. Highly mobile and associated with plasmids, they are prevalent in water sources due to extensive tetracycline use in agriculture [[10], [11], [12]]. Of the two, *tetA* is reported to be more prevalent making it an ideal anthropogenic pollution marker [12,13] although regional and environmental variations can influence its prevalence. *ermF* and *qnrS,* responsible for macrolide and quinolone resistance respectively, are often associated with mobile genetic elements, facilitating their spread across clinical and environmental matrices [12]. Clinically significant ARGs like *blaTEM* (β-lactam resistance) [12] and *mecA* (methicillin resistance in MRSA) [14] are widespread in wastewater, reflecting their healthcare-associated antibiotic use and illustrating the environmental dissemination of clinically derived ARGs. Notably, *mcr-1,* which confers resistance to colistin, a last-resort antibiotic, presents a critical threat due to its rapid dispersion potential, despite its rare detection [12]. Aminoglycoside resistance genes *aadA* [11] and *aph(3′')-Ib* [15,16] are similarly widespread in clinical environments and wastewater, signifying both clinical relevance and environmental persistence. These genes are further classified under WHO’s access (e.g., *aadA, aph(3″)-Ib, tetA, tetB, blaTEM*), watch *(*e.g., *mecA, ermF, qnrS*)*,* and reserve (e.g., *mcr-1*) antibiotic groups [8]. These targets complement an already established set of ARGs, including *sul1, sul2, tetM, tetW,* and the anthropogenic AMR indicator *intI1* [17,18] that have been widely used in the studies on environmental AMR. Primers, probes and gblock sequences of *sul1, sul2, tetW, tetM,* and *intI1* are presented in supplementary Table 2.

Together, these assays provide a methodologically unified quantification approach for of a set of targets across a broad set of resistance classes, and a range of abundance and clinical importance, enabling a wide range of applications. The detection and quantification of these ARGs in environmental matrices could highlight the current resistance landscape and allow linking specific anthropogenic sources and origin. This enables the assessment of risk associated with the spread of clinically important resistance genes into the environment, highlighting their role in the broader issue of AMR and guiding risk mitigation strategies.

### Primer, probe, and gBlock design and in silico validation

Neither degenerate nor majority rule was applied in primers, probes and gBlocks design; rather we used a conserved rule approach. The updated ARG target sequences viz., *aph(3″)-Ib* (ARO:3002639), *ermF* (ARO: 3000498), *tetA* (ARO:3000165), and *tetB* (ARO:3000166) were retrieved from Comprehensive Antibiotic Resistance Database (CARD, https://card.mcmaster.ca/, Accessed on 07.12.2023) to design primers and probe sequences targeting conserved regions of the alignment. For *aadA*, retrieved sequence ARO:3002601 was used to design a probe for the existing primer pairs [18]. Primers and probe were designed in Geneious prime (Version 2023.2.1), utilizing Primer3 (Version 2.3.7) with default settings.

To accommodate and represent genetic diversity within target genes, multiple sequence alignments were performed with CLUSTAL Omega 1.2.2 utilizing Geneious prime (Version 2023.2.1), for the ARG variants retrieved from CARD (https://card.mcmaster.ca/, Accessed on 07.12.2023). Following the alignment, a consensus sequence was generated to reflect the common features of the aligned ARG variants, using consensus sequence generation tool in Geneious prime (Version 2023.2.1). A consensus alignment of variants was constructed to validate the existing primers and probes for *aadA, blaTEM, mcr-1,* and *qnrS.* The alignment included *aadA1* and *aadA2; blaTEM-1, blaTEM-2, blaTEM-3, blaTEM-5, blaTEM-6, blaTEM-7, blaTEM-9, blaTEM-10, blaTEM-12, blaTEM-35, blaTEM-135,* and *blaTEM-150; mcr-1.1 to mcr-1.13, mcr-1.18, mcr-1.19, mcr-1.22, mcr-1.26, mcr-1.27, mcr-1.32, mcr-1.33,* and *mcr-1.34;* and *qnrS1, qnrS2, qnrS3, qnrS4, qnrS5, qnrS6, qnrS8, qnrS9, qnrS10, qnrS11, qnrS12,* and *qnrS15.* The alignment served to ensure the primers and probes, as described in previous studies for *aadA* [19], *blaTEM* [20], *mcr-1* (see Table 1), and *qnrS* [21], effectively targeted the diverse genetic variants. All primers and probes target conserved regions of the alignments. Specific gene variants were chosen based on the gene prevalence, and availability of published literature as described in CARD (https://card.mcmaster.ca/) and availability of test strains (e.g., *blaTEM-35, blaTEM-135, blaTEM-150*). For mecA, the existing primers and probe [22] were validated with *mecA* sequence (ARO:3000617) retrieved from CARD. Corresponding CARD accession no and consensus alignments are provided supplementary Table 1. The conserved primers, and probe sequences used for detection and qPCR product sizes are presented in Table 1.

**Table 1:** List of primers and probe sequences.

ARG	Primer / Probe	Sequence 5′−3′	Reference	Product size (bp)
*mcr-1*	mcr-1-F	GGGCCTGCGTATTTTAAGC	This study*	164
	mcr-1-R	TGCAGCCACTGGATACTTTG		
	mcr-1-Probe	5′-FAM-CGTGAATTT-ZEN-GGCAAACTTTT-3′-IABkFQ		
*mecA*	mecA-F	CATTGATCGCAACGTTCAATTTAAT	[22]	99
	mecA-R	TGGTCTTTCTGCATTCCTGGA		
	mecA-Probe	5′-FAM-CTATGATCC-ZEN—CAATCTAACTTCCACATACC-3′-IABkFQ		
*aadA*	aadA-F	GTTGTGCACGACGACATCATT	[19]	101
	aadA-R	GGCTCGAAGATACCTGCAAGAA		
	aadA- Probe	5′-FAM-AATTTGGAG-ZEN-AATGGCAGCGC-3′-IABkFQ	This study	
*aph(3″)-lb*	aph(3″)-lb-F	ACCGGACGAGGACAAGAGTA	This study	134
	aph(3″)-lb-R	CCATGAAGTTCGGCATGCAG		
	aph(3″)-lb-Probe	5′-FAM-AGCTCGATC-ZEN-TTTTGGCTCGT-3′-IABkFQ		
*tetB*	tetB-F	ATCTTTGCTCCTTGGCTTGGA	This study	150
	tetB-R	CCCTGAAAGCAAACGGCCTA		
	tetB- Probe	5′-FAM-CTGGCTTTT-ZEN-TCAAGTGCGCT-3′-IABkFQ		
*tetA*	tetA-F	CTATATCGGGCGGATCGTGG	This study	84
	tetA-B	TCGCCATCAGTGATATCGGC		
	tetA- Probe	5′-FAM-CGGTAGCCG-ZEN-GCGCTTATATT-3′-IABkFQ		
*ermF*	ermF-F	TCGTTTTACGGGTCAGCACT	This study	119
	ermF-R	TAAGAAACCCCTTGCCTGCC		
	ermF- Probe	5′-FAM-AGCAAATAT-ZEN-AAGTAATCAGGATACGGT-3′-IABkFQ		
*blaTEM*	blaTEM-F	CACTATTCTCAGAATGACTTGGT	[20]	85
	blaTEM-R	TGCATAATTCTCTTACTGTCATG		
	blaTEM- Probe	5′-FAM-CCAGTCACA-ZEN-GAAAAGCATCTTACGG-3′-IABkFQ		
*qnrS*	qnrS-F	GACGTGCTAACTTGCGTG	[21]	118
	qnrS-R	TGGCATTGTTGGAAACTT		
	qnrS- Probe	5′-FAM-TACGACATT-ZEN—CGTCAACTGCAAGT-3′-IABkFQ		

⁎ The primers and probe of *mcr-1* was shared by Dr. Xavier Bellanger (LCPME UMR7564 CNRS-Université de Lorraine) designed for the MicrobiEAU project funded by the Université de Lorraine (LUE programme).

**Fig. 1:**
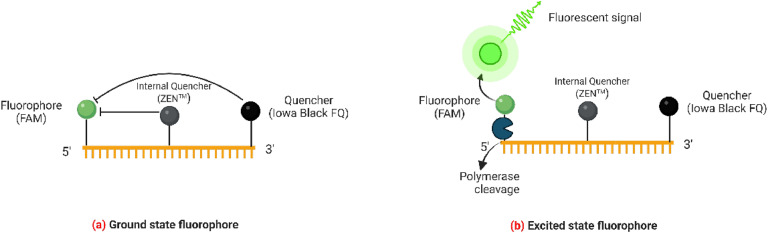
Representation of (a) ground state and (b) excited state of double quenched TaqMan probe.

Double-quenched TaqMan probes, e.g., *mcr-1* Probe: 5′-FAM-CGTGAATTT-ZEN-GGCAAACTTTT-3′-IABkFQ (Table 1), designed and developed by Integrated DNA Technologies, Inc is expected to enhance the sensitivity and specificity in qPCR assays. In this probe, FAM represents a 5′ fluorophore (FAM), ZEN is an internal quencher positioned within the sequence, and IABkFQ (Iowa Black FQ) is a 3′ quencher (Fig. 1a). The presence of both the ZEN internal quencher and the 3′ Iowa Black FQ quencher improves the quenching efficiency, minimizing background fluorescence when the probe is intact.

During amplification, the probe binds to the target sequence. As the DNA polymerase extends the primers, it cleaves the probe, separating the FAM fluorophore from the quenchers, resulting in a fluorescence signal proportional to the amount of target ARG (Fig. 1b). This dual-quenching design reduces non-specific signal, leading to increased accuracy, sensitivity, and precision in detecting low-abundance targets or targets in complex samples. The probe design also helps in shortening the probe length while maintaining quenching efficiency, thus providing better performance in qPCR assays [23]. Probe sequences without the fluorescent dye (FAM) at the 5′ end, the internal quencher (ZEN), and the 3′ quencher (IABkFQ) are presented in supplementary Table 2.

The updated target ARG sequences viz., *aadA* (ARO:3002601), *aph(3″)-Ib* (ARO:3002639), *blaTEM* (ARO:3000873), *ermF* (ARO: 3000498), *mecA* (ARO:3000617), *mcr-1* (ARO:3007230), *qnrS* (ARO: 3004627), *tetA* (ARO:3000165), and *tetB* (ARO:3000166) were retrieved from CARD to design gBlocks for standard curve generation. The gBlock standards were designed as libraries, each library consisting of up to 5 ARGs. These gBlock libraries include the entire target region amplified by both primers and probe, with a 6 bp flanking sequence (5′-GAATTC-3′) to ensure proper binding of the primers [24]. Including a standardized flanking sequence such as 5′-GAATTC-3′ (EcoR1 restriction site), could minimize the risk of amplification errors or PCR artifacts, thereby improving assay specificity and reliability. The flanking regions also provides flexibility to design standards for multiple genes, allowing incorporation of several genes into a single library enabling development of multiplex assays, while maintaining the integrity of each target. These regions provide space for primers to bind without interfering with the core gene sequence of adjacent genes in gBlock libraries. The gBlock libraries (gBlock1_mcr1_mecA_aadA_aph3Ib_tetB and gBlock2_tetA_ermF_blaTEM_qnrS) used for standard curve construction are presented in Table 2. The gBlock sequence of individual genes, without the flanking regions, are presented in supplementary Table 2.

**Table 2:** List of gBlock libraries used as qPCR standards and the library sequence. Flanking regions (3′-GAATTC-5′) are marked in lowercase letters, target regions of specific ARGs are in the order shown in the gBlock name.

gBlock	Sequence 5′ - 3′
gBlock1_mcr1_mecA_aadA_aph3Ib_tetB	gaattcGGGCCTGCGTATTTTAAGCGATATGATGAAAAGTTTGCCAAATTCACGCCAGTGTGTGAAGGTAATGAGCTTGCCAAGTGCGAACATCAGTCCTTGATCAATGCTTATGACAATGCCTTGCTTGCCACCGATGATTTCATCGCTCAAAGTATCCAGTGGCTGCAgaattcCATTGATCGCAACGTTCAATTTAATTTTGTTAAAGAAGATGGTATGTGGAAGTTAGATTGGGATCATAGCGTCATTATTCCAGGAATGCAGAAAGACCAgaattcGTTGTGCACGACGACATCATTCCGTGGCGTTATCCAGCTAAGCGCGAACTGCAATTTGGAGAATGGCAGCGCAATGACATTCTTGCAGGTATCTTCGAGCCgaattcACCGGACGAGGACAAGAGTACGCCGCAGCTCGATCTTTTGGCTCGTGTCGAACGAGAGCTACCGGTGCGGCTCGACCAAGAGCGCACCGATATGGTTGTTTGCCATGGTGATCCCTGCATGCCGAACTTCATGGgaattcATCTTTGCTCCTTGGCTTGGAAAAATGTCTGACCGATTTGGTCGGCGCCCAGTGCTGTTGTTGTCATTAATAGGCGCATCGCTGGATTACTTATTGCTGGCTTTTTCAAGTGCGCTTTGGATGCTGTATTTAGGCCGTTTGCTTTCAGGGgaattc
gBlock2_tetA_ermF_blaTEM_qnrS	gaattcCTATATCGGGCGGATCGTGGCCGGCATCACCGGGGCGACTGGGGCGGTAGCCGGCGCTTATATTGCCGATATCACTGATGGCGAgaattcTCGTTTTACGGGTCAGCACTTTACTATTGATAAAGTGCTAATAAAAGATGCAATAAGACAAGCAAATATAAGTAATCAGGATACGGTTTTAGATATTGGGGCAGGCAAGGGGTTTCTTAgaattcCACTATTCTCAGAATGACTTGGTTGAGTACTCACCAGTCACAGAAAAGCATCTTACGGATGGCATGACAGTAAGAGAATTATGCAgaattcGACGTGCTAACTTGCGTGATACGACATTCGTCAACTGCAAGTTCATTGAACAGGGTGATATCGAAGGCTGCCGCTTTGATGTCGCAGATCTTCGTGATGCAAGTTTCCAACAATGCCAgaattc

### DNA extraction

Clinical strains, which are whole genome sequenced and verified to either harbor ARG variants (positive strain control DNA, PSC), and strains confirmed to lack the target genes (negative strain control DNA, NSC), were obtained in sheep blood agar plates from the Applied Microbiology Research Lab, Institute of Medical Microbiology, University of Zurich. The strains were processed in a BSLII laboratory following standard operating procedure for handling clinical pathogens carrying ARGs. A summary of the test strains is presented in Table 3. Genomic DNA from a few colonies of the reference strains was extracted in duplicate using QIAmp® DNA Mini kit (Qiagen, Germany) following the manufacturer’s instruction and stored at −20 °C for downstream analysis.

**Table 3:** List of clinical strains used for primers and probe validation.

ARG variants tested	Reference strain	Internal reference*	Expected	Experimental validation (Mean Ct of 4 replicates)
*aadA1*	*Acinetobacter baumannii*	5f5a9457	+	+ (19.97)
*aadA1*	*Acinetobacter baumannii*	e70321e7	–	–
*aadA2*	*Enterobacter cloacae*	743a5edb	+	+ (24.72)
*aadA2*	*Enterobacter cloacae*	c6ec50ff	–	–
*aph(3′')-Ib*	*Pseudomonas aeruginosa*	9afcc077	+	+ (18.03)
*blaTEM-1*	*Escherichia coli*	0259bf71	+	+ (18.40)
*blaTEM-1*	*E. coli*	6961107c	–	–
*blaTEM-12*	*Acinetobacter baumannii*	aa4e92ba	+	+ (20.76)
*blaTEM-12*	*Acinetobacter baumannii*	e70321e7	–	–
*blaTEM-135*	*Klebsiella pneumoniae*	b7f02bd9	+	+ (17.48)
*blaTEM-135*	*Acinetobacter baumannii*	e70321e7	–	–
*blaTEM-150*	*Klebsiella pneumoniae*	409df3b6	+	+ (19.68)
*blaTEM-150*	*Klebsiella pneumoniae*	600819d2	–	–
*blaTEM-35*	*E. coli*	569ba05d	+	+ (19.01)
*blaTEM-35*	*E. coli*	6961107c	–	–
*ermF*	*Acinetobacter baumannii*	aa4e92ba	–	–
*mcr-1.8*	*E. coli*	dbde13a4	+	+ (17.45)
*mcr-1.8*	*E. coli*	0259bf71	–	–
*mecA*	*Staphylococcus aureus*	00e009e5	+	+ (18.01)
*mecA*	*E. coli*	7d243655	–	–
*qnrS1*	*E. coli*	01eb6b0b	+	+ (20.26)
*qnrS1*	*E. coli*	0259bf71	–	–
*qnrS13*	*E. coli*	6961107c	+	+ (24.30)
*qnrS13*	*E. coli*	0259bf71	–	–
*tetA*	*Enterobacter cloacae*	743a5edb	+	+ (20.03)
*tetA*	*Enterobacter cloacae*	c6ec50ff	–	–
*tetB*	*E. coli*	1b764e79	+	+ (18.30)
*tetB*	*E. coli*	0259bf71	–	–

⁎ The internal reference code of Applied Microbiology Research Lab, Institute of Medical Microbiology, University of Zurich.

DNA extracted from treated effluent of two wastewater treatment plants viz., ARA Falkenstein, and STEP-Ville de Lausanne, Switzerland was used to validate the designed primes/probes. Duplicate grab samples were collected in 5 L sterile sampling bottles. The sampling bottles were stored at 4 °C in the dark during transport to the lab and prior to processing within 24 h of collection. Biomass from the collected wastewater samples were filter-concentrated using sterile 0.22 µm, 47 mm cellulose ester S-Pak filter (Merck, Germany) until the pores were clogged. The filters were then stored at −80 °C prior to DNA extraction. DNA was extracted from the filters using the DNeasy Power Water Kit (Qiagen, Germany) following the manufacturer’s instruction and stored at −20 °C. All frozen extracts from isolates and wastewater samples were thawed on ice prior to downstream analysis.

The integrity of the extracted DNA was checked on an agarose gel. The quality (260/280 and 260/230 absorbance ratios) of the extracted DNA was determined using NanoDrop One Spectrophotometer (Thermo Fischer Scientific, USA). Qubit™ 4 Fluorometer (Thermo Fisher Scientific, USA) was used to quantify DNA concentration using the Qubit™ dsDNA BR Assay Kit, following the manufacturer's instructions. Extracts of sufficient DNA concentration and purity for evaluation of primer set and probe performance were obtained (Supplementary Table 3 and 4).

## Resuspension and dilution of gBlock libraries

Dry gBlock gene libraries (Integrated DNA Technologies, Belgium) were resuspended in Poly A (0.5 mg/mL, Merck, Switzerland) prepared in buffer AE (Elution buffer, Qiagen, Germany) to yield a concentration of 10 ng/µL following the manufacturer’s recommended procedures and guidelines. Poly A prevents loss of material due to adherence to the storage tubes when the gBlock concentration is <1 ng/µL, by acting as a carrier [25]. The concentration of the resuspended gBlocks were verified using NanoDrop One Spectrophotometer (Thermo Fischer Scientific, USA) to be 10 ng/µL.

Copy number of the resuspended gBlocks in copies/µL was calculated according to Eq. (1) [25].

(1) $$\frac{\text{Concentration}\text{of}\text{gBlock}\text{fragment}\text{in}\;\frac{ng}{\mu L}\times \;\text{Molecular}\text{weight}\text{in}\text{fmol}\;/ng\times \;{\text{Avogadro}}^{\prime }s\text{number}}{1\times 10^{15}fmol/mol}$$

Based on the calculated copy number, individual gBlocks were diluted with Poly A (0.5 mg/mL) to obtain an initial concentration of $25\;\times \;10^{7}$copies/µL. Aliquots of 10 µL stock were stored in nuclease-free PCR tubes (Nippon Genetics, Europe) at −20 °C prior to use. The gBlocks were thawed on ice and serially diluted to 25 copies with nuclease-free molecular grade water (Sigma-Aldrich, UK) when required and stored at 4 °C for up to two weeks.

### qPCR assay and optimization

All qPCR reagents were stored as per manufacturer recommendations and thawed on ice when required. The reaction mix containing all reagents except the gBlocks was prepared per manufacturer’s recommendation in a laminar flow cabinet under sterile condition. The qPCR assays were performed in triplicates and quantification cycle (Ct) values were determined on the LightCycler®480 Roche (Roche, Switzerland) real-time qPCR platform and software (Version 1.5.1.62 SP3). The final volume of the qPCR reaction mix was 10 µL (Table 4), comprising 5 µL of LightCycler® 480 Probes Master (2x concentration, final concentration 1x), 2 µL of gBlock library (25 × 10 ^7^– 25 copies/µL), and varying volumes of primers, probes, and LightCycler® 480 Probes Master H_2_O, depending on the specific reaction requirements. The optimized primers and probe volume varied between 0.03 and 0.04 μL per reaction. The final volume was adjusted to 10 µL with LightCycler® 480 Probes Master H_2_O [26].

**Table 4:** qPCR reaction setup.

Reagents	Stock conc.	Final conc.	Volume per reaction (µL)
LightCycler® 480 Probes Master (X)	2	1	5
Forward primer (μM)	100	0.3 – 0.4	0.03 – 0.04
Reverse primer (μM)	100	0.3 – 0.4	0.03 – 0.04
TaqMan probe (μM)	100	0.3 – 0.4	0.03 – 0.04
LightCycler® 480 Probes Master H_2_O (µL)	–	–	2.91 – 2.88
gBlock (copies/µL)	$25\;\times \;10^{7\;}-25$	$50\;\times \;10^{7}-50$	2
Reaction volume (µL)	–	–	10

The primers and probe concentrations ranging from 0.25 - 0.45 μM were tested to identify the concentration that yielded amplification efficiency above 90 %. The optimized concentration for each primer pair and probe is presented in Table 5.

**Table 5:** Optimized TaqMan assay conditions.

Primer / Probe	Concentration (μM)	Annealing temperature ( °C)	Annealing time (*sec*)	Product size (bp)	Amplification efficiency ( %)
mcr-1-F	0.03	60	15	164	92.29
mcr-1-R					
mcr-1-Probe					
mecA-F	0.03	60	45	99	91.40
mecA-R					
mecA-Probe					
aadA-F	0.03	60	15	101	94.81
aadA-R					
aadA- Probe					
aph(3′')-lb-F	0.03	60	15	134	96.70
aph(3′')-lb-R					
aph(3′')-lb- Probe					
tetB-F	0.03	60	15	150	94.81
tetB-R					
tetB- Probe					
tetA-F	0.03	62	15	84	91.80
tetA-B					
tetA- Probe					
ermF-F	0.035	60	15	119	95.92
ermF-R					
ermF- Probe					
blaTEM-F	0.035	60	15	85	94.17
blaTEM-R					
blaTEM- Probe					
qnrS-F	0.04	60	15	118	91.78
qnrS-R					
qnrS- Probe					

**Fig. 2:**
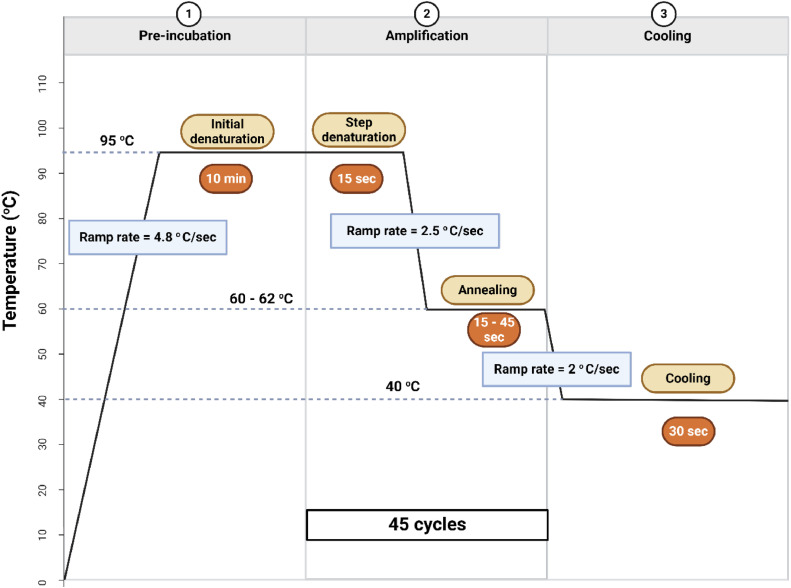
Thermal cycling conditions.

To optimize the reaction conditions, a range of annealing temperature (56 – 62) °C and annealing time (15 – 45 s) settings were tested. Table 5 presents the optimized annealing temperature and annealing time for each ARG. The thermal cycling conditions of the reaction are as follows: Pre-incubation @ 95 °C (ramp rate = 4.8 °C/*sec*) for 10 min, followed by 45 cycles of amplification, which includes step denaturation @95 °C (ramp rate = 4.8 °C/*sec*) for 15 s, annealing @ 60 – 62 °C (ramp rate = 2.5 °C/*sec*) for 15–45 s, and extension @72 °C (ramp rate = 4.8 °C/*sec*) for 01 s followed by cooling @ 40 °C (ramp rate = 2 °C/*sec*) for 30 s [26]. The extension step was not included in the assays, as prior experiments showed no significant improvement in amplification efficiency or copy number accuracy when included (Fig. 2). The thermal cycling conditions including time and ramp rate are per manufacture’s recommendations.

### Limit of quantification, limit of detection and qPCR efficiency

As a part of the consolidated method, we propose an updated procedure based on our previous method in [18], for determining quality standards for qPCR quantification and defining a limit of quantification and limit of detection for gene quantification in environmental samples.

This approach assumes that all qPCR reactions are run with a minimum of three replicates (standards, negative controls, and samples) allowing calculation of the standard deviation of the Ct. Standard dilutions of gBlock standards are prepared over at least 7 orders of magnitude (5 × 10^7^ to 50 copies / reaction) and are included in every run. A standard curve of the form Ct = *m*(log_10_(copies)) + *b* is calculated by linear regression, where m and b are the slope and intercept from the standard curve.

To qualify for evaluation, the coefficient of determination (R²) of the standard curve must be greater than 0.99.

In addition, the efficiency (E) calculated according to Eq. (3) must be between 90 and 110 %.

(3) $$E=\left(10^{\left(-\frac{1}{m}\right)}-1\right)\times \;100\%$$

We consider an error threshold (Eq. (4)), where the standard deviation of the replicate Ct values (σ_rep_) of a sample > 10 % of the Ct difference between log units (= slope, m) is deemed unreliable for quantification. Samples with significant deviation are repeated, and those remaining unreliable are flagged with a (U).

(4) $$\;\text{Error}\text{threshold}\;=\sigma_{rep}>\left|0.1\cdot m\right|$$

The error threshold can be adjusted to other values based on the specific requirements of an analysis, but we recommend keeping it in the range of 0.1–1 in terms of absolute values or 1–10 % in terms of coefficient of variation (CV).

To further minimize potential errors, multiple dilutions (e.g., 1:10, 1:100, 1:100) of sample DNA can be evaluated to identify the optimal dilution with reduced inhibition. Apparently increasing copy numbers in higher dilutions (after correcting for the dilution factor) indicate the presence of inhibitors, and more diluted samples are to be preferred. To select the optimal dilution from the tested range, check for consistent Ct values across replicates and between the dilutions with minimal standard deviation (i.e., CV ≤ 5 %). Exclude dilutions exceeding the CV > 5 %, as they indicate unreliable variability.

We define the lower limit of calibration (LLC, Eq. (5)) as the average Ct of the most dilute standard (MDS) that passes the error threshold (i.e., after unreliable dilution levels are removed).

(5) $$LLC={\bar{Ct}}_{MDS}$$

The limit of detection (LOD) under consideration of the error threshold is set to the lowest recorded Ct of any individual negative control sample minus error threshold (|0.1· m|) (Eq. (6.1)).

(6.1) $$LOD_{negatives}=lowest\left(Ct_{neg}\right)--\left(\left|0.1\cdot m\right|\right)$$

Or the intercept of the standard curve (expected Ct of 1 gene copy) minus |0.1· m| (Eq. (6.2)).

(6.2) LODintercept=b−−|0.1·m|

whichever is the lower value, i.e., if LOD_negatives_ < LOD_intercept_, then LOD = LOD_negatives_; otherwise LOD = LOD_intercept_.

Samples with LOD > $\bar{\text{Ct}}\;$> LLC are considered quantifiable by extrapolation, if they fulfill the low error of replicates condition σ_rep_ ≤ |0.1· m| and are within one log unit of the LLC (i.e., $\bar{\text{Ct}}\;$≤ LLC +|m|). This condition also defines the limit of quantification (LOQ) in Eq. (7) as:

(7) LOQ=LLC+|m|

If LOD < LOQ, then LOQ = LOD. In this case, the samples quantified by extrapolation are marked “E”.

LOQ and LOD are here defined in terms of acceptable Ct values. These can be converted into copy numbers (per reaction) using the standard curve (LOQ_C_, LOD_C_) or environmental concentrations (such as copies/mL or copies/g) in analogy to the calculations as performed for samples, stating the assumed dilution factor, DNA extraction volume and sample volume/mass. These values are designated LOQ_E_ and LOD_E_, respectively.

## Method validation

### Interpretation of qPCR standard plots and test results

#### Efficiency, analytical sensitivity, and intra-assay variability

gBlock at $25\;\times \;10^{7}$copies/µL concentration, serially diluted (5 × 10^7^ – 50 copies/reaction) was used to construct standard curves.

**Fig. 3:**
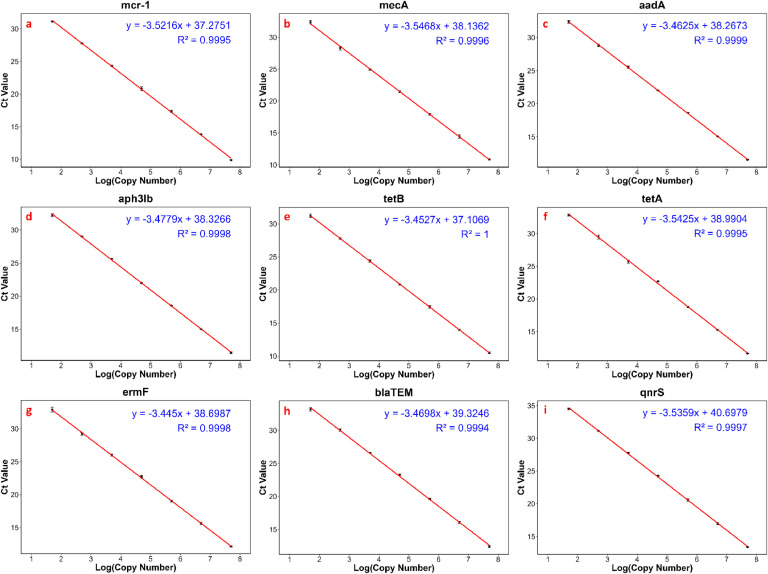
qPCR standard curves for (a) *mcr-1*, (b) *mecA*, (c) *aadA*, (d) *aph(3″)-Ib*, (e) *tetB*, (f) *tetA*, (g) *ermF*, (h) *blaTEM*, and (i) *qnrS.*

The slope and coefficient of determination (R^2^) were obtained from the standard plot (Ct versus the log of the respective diluted gBlock concentrations). The observed R^2^ > 0.99 reflects a strong linear relationship between the log concentration of the standard dilutions and the Ct values (Fig. 3).

No amplification (Ct_neg_ = 0) was observed for negative controls (NEG), resulting in LOD_negatives_ = 0. The intercept of the standard curve was used to calculate LOD_intercept_ for each assay, making LOD = LOD_intercept_ for all assays. Consistency of amplification can be confirmed by Ct values with higher gBlock concentration producing lower Ct values and vice versa, across standards and qPCR assays (Table 6). The intra-assay precision was ensured by performing a minimum of three replicates per run, while inter-assay precision was confirmed by performing a minimum of three runs. The assay demonstrated high reproducibility, with minimal variability between technical replicates (σ_rep_ < 0.5) and inter-assay variability (CV < 5 %) across independent runs, consistently observed at each concentration In summary, all the assays showed good intra- (σ_rep_ < 0.5) and inter- assay repeatability (CV < 5 %), LLC (50 copies µL^-1^), LOQ, LOD, amplification efficiency (91.40 – 95.92 %), linearity (R^2^ > 0.99), and overall calibration range (9.88 – 34.51) (Table 6).

**Table 6:** Amplification efficiency, coefficient of determination (R^2^), calibration range, lower limit of calibration (LLC), Limit of detection (LOD), and limit of quantification (LOQ) of the assays.

ARG	Slope	Amplification efficiency ( %)	R^2^	Calibration range of 5 × 10^7^ to 50 copies (Ct)	LLC (Ct)	LOQ (Ct)	LOD (Ct)
*mcr-1*	−3.5216	92.29	0.9995	9.88 – 31.13	31.13	34.65	36.92
*mecA*	−3.5468	91.40	0.9996	10.84 – 32.36	32.36	35.91	37.78
*aadA*	−3.4625	94.45	0.9999	11.55 – 32.39	32.39	35.85	37.92
*aph(3′')-lb*	−3.4779	93.88	0.9998	11.46 – 32.24	32.24	35.72	37.98
*tetB*	−3.4527	94.81	1.0000	10.50 - 31.22	31.22	34.67	36.76
*tetA*	−3.5425	91.55	0.9995	11.63 – 32.87	32. 87	36.41	38.64
*ermF*	−3.4450	95.92	0.9998	12.12 – 32.90	32.90	36.35	38.35
*blaTEM*	−3.4698	94.17	0.9994	12.42 – 33.17	33.17	36.64	38.98
*qnrS*	−3.5359	91.78	0.9997	13.36 – 34.51	34.51	38.05	40.34

## Experimental validation with controls

The analytical specificity of the assays was confirmed with DNA extracted from strains carrying different variants of the target ARG (PSC), those not harboring the target ARGs (NSC) and herring sperm DNA (general no target control, NTC) (Table 3). We tested for the presence of reagent contamination or self-priming by including negative controls (NEG, using molecular grade water, Sigma-Aldrich, UK instead of sample) and extraction blank controls (BLK). Assay performance on the target ARGs in a complex environmental matrix was evaluated using DNA extracted from two different wastewater effluents. The Ct values of the tested PSC, NSC and environmental DNA are provided in supplementary Table 3 and 4.

All NEGs and BLKs showed no amplification (no Ct detected, i.e., Ct > 45) showing the absence of unintended contamination with amplifiable material. We consistently observed a lack of detectable amplification (Ct = 0) for all controls lacking the targeted genes (NSC and NTC) demonstrating the specificity of the primers and probes in the developed qPCR assays.

On the other hand, test controls carrying the target ARGs (PSC) consistently showed robust amplification, confirming the sensitivity and accuracy of the assays. Elution volume and filtration volume used to determine ARG concentration in copies/mL are presented in supplementary Table 4.

To decide on the best dilution for quantification and reporting, the Ct values of replicates in each dilution against the calibration range of the gene target can be considered. E.g., for *aadA* quantification in effluent from ARA-Falkenstein, both the 1:10 (Ct = 26.6) and 1:100 (Ct = 29.4) dilutions fall well within the calibration range of Ct (11.55 – 32.39) of the assay with CV = 0.43 % and 0.72 %, respectively indicating effective amplification without significant inhibition. In this case, the 10-fold dilution is particularly sensitive and ideal for detecting and quantifying when *aadA* is expected to be low in abundance, making it a strong choice for primary quantification. Meanwhile, the 100-fold dilution can serve as a quality control, verifying consistency across replicates and providing assurance that inhibitors do not significantly affect amplification efficiency or target quantification. In case of *blaTEM,* between 1:10 (Ct = 32.44, CV = 0.26 %) and 1:100 (Ct = 34.10, CV = 2.2 %), 1:10 dilution can be chosen as it fell within the calibration range of Ct (12.42 – 33.17) of the assay. In any case, employing both or more dilutions could strategically enhance the assay’s reliability and accuracy, especially in complex environmental samples. The average Ct values for *blaTEM* (1:100), *tetB* (1:10), and *tetB* (1:100) for ARA-Falkenstein and *aph(3″)-Ib* (1:100), and *mcr-1* (1:10) for STEP-Ville de Lausanne are considered unreliable (U) as they do not meet the error threshold (σ_rep_ ≤ |0.1· m|) criterion as outlined in the “Limit of quantification, limit of detection and qPCR efficiency” sub-section. Detailed calculation for all criteria: error threshold (σ_rep_ ≤ |0.1· m|)), out of calibration (Avg. Ct ≤ LLC), quantifiable range (Avg. Ct ≤ LOQ) and detectability (Avg. Ct > LLOD) are presented in supplementary Table 4.

Overall, the results indicate that the assays effectively detected multiple ARGs across different sample types and dilution factors (Table 7). For example, in the effluent metagenome of STEP-Ville de Lausanne, Switzerland, the gene *aadA* exhibited an average Ct of 21.64 with 6.83 × 10^4^ gene copies/reaction at 1:10 dilution, yielding an ARG concentration of 9.95 × 10^4^ copies/mL. Similarly, the gene *ermF* showed a robust performance with an average Ct of 21.29 and an ARG concentration of 1.89 × 10^5^ copies/mL at 1:10 dilution*. aph(3″)-Ib* gene displayed varying detection and quantification levels based on dilution factors, with concentrations ranging from 4.16 × 10^4^ and 6.39 × 10^4^ copies/mL at 1:10 and 1:100 dilution. The assay also demonstrated sufficient sensitivity for the detection of low-abundance genes, such as *mcr-1,* which reported a Ct of 34.90 and 36.89 (<LOD_mecA_, Ct = 36.92) at 1:10 and 1:100 dilution, respectively. The Ct values for *aph(3″)-Ib* (Ct =25.82) at 1:100 dilution and mecA (Ct = 36.97) at 1:10 dilution were deemed unreliable (U) as they failed to meet the error threshold (σ_rep_ ≤ |0.1· m|)) (Table 7). Notably, the *qnrS* gene was not quantifiable in environmental samples although a positive control successfully yielded amplification. Despite its prevalence, the absence of *qnrS* in the studied samples may be attributed to the low abundance of the gene influenced both by regional distribution and prevalence [13]. Further, environmental samples without the target genes further confirm the assay’s specificity, as no amplification was observed under any condition (Table 7). The differences in gene detection across wastewater treatment plants highlight the critical need for optimizing qPCR assays when analyzing complex environmental samples. It is important to account for sample-specific variability such as inhibitors, microbial diversity, and matrix complexity to ensure reliable detection of target genes. Furthermore, effective optimization can improve the sensitivity of the assays, allowing for the detection of low-abundance genes. Overall, the findings confirm the reliability and applicability of the developed qPCR methods for monitoring ARGs in wastewater matrices.

**Table 7:** qPCR results of environmental test controls.

Sample	Wastewater treatment plant	ARG	Average Ct	Dilution factor	ARG conc. (Copies/mL)	Reliability
Effluent	ARA-Falkenstein	*aadA*	26.6	10	3.96E+03	(R)
			29.4	100	6.04E+03	(R)
		*aph(3″)-Ib*	26.6	10	2.00E+03	(R)
			30.1	100	2.78E+03	(R)
		*blaTEM*	32.4	10	5.24E+01	(R)
			34.1 (>LLC)	100	1.15E+02	(U)
		*ermF*	26.9	10	4.87E+03	(R)
			30.2	100	3.88E+02	(R)
		*mcr-1*	- (>LOD)	10	-	**–**
			- (>LOD)	100	-	**–**
		*mecA*	- (>LOD)	10	-	**–**
			- (>LOD)	100	-	**–**
		*qnrS*	- (>LOD)	10	-	**–**
			- (>LOD)	100	-	**–**
		*tetA*	28.8	10	8.05E+02	(R)
			32.6	100	6.60E+02	(R)
		*tetB*	33.6 (>LLC)	10	1.37E+01	(U)
			35.9 (<LOD)	100	2.22E+01	(U)
Effluent	STEP-Ville de Lausanne	*aadA*	21.6	10	9.95E+04	(R)
			25.1	100	1.03E+05	(R)
		*aph(3″)-Ib*	22.4	10	4.16E+04	(R)
			25.8	100	6.39E+04	(U)
		*blaTEM*	26.9	10	5.05E+03	(R)
			30.2	100	3.83E+03	(R)
		*ermF*	21.3	10	1.89E+05	(R)
			24.8	100	2.18E+05	(R)
		*mcr-1*	34.9 (<LOD)	10	1.03E-06	(R)
			36.9 (<LOD)	100	3.18E-10	(R)
		*mecA*	37.0 (<LOD)	10	1.18E+00	(U)
			- (>LOD)	100	-	-
		*qnrS*	- (>LOD)	10	-	-
			- (>LOD)	100	-	-
		*tetA*	24.7	10	1.18E+04	(R)
			27.0	100	2.58E+04	(R)
		*tetB*	25.5	10	3.03E+03	(R)
			28.8	100	3.23E+03	(R)

*LLC: Lower limit of calibration, LOD: Limit of detection, (U): Unreliable, (R): In range.

### Limitations

TaqMan probe-based qPCR methods for detecting ARGs in environmental samples may face several challenges. Environmental matrices like soil or water often contain inhibitors, such as humic acids, heavy metals, and organic compounds, which can interfere with the PCR reaction, reducing efficiency and sensitivity, leading to false negatives, or underestimation of the target abundance [27]. DNA extraction from complex samples is also challenging due to contamination, low yields, or heterogeneous microbial communities, affecting detection accuracy. Low-abundance targets may fall below the detection limit, while the presence of vast microbial diversity increases the risk of non-specific amplification and cross-reactivity, causing false positives or overestimations. To overcome these limitations, use of suitable extraction and cleanup protocol that minimize the inhibitors, use of inhibitor-resistant reagents, effective dilution of inhibitors, careful optimization of DNA extraction to increase the yield, and increasing sample volumes can improve sensitivity and enhance the accuracy of absolute quantification, ultimately improving the reliability of qPCR for effective surveillance. Additionally, incorporating internal standards or spike-in controls in the experiment design can aid in monitoring the extent of inhibition during the qPCR process, ensuring more accurate quantification, and reducing the likelihood of false negatives or underestimation of target abundance [28]. However, in this regard, the TaqMan assay has considerable advantages over the qPCR assay designs that do not employ a specific reporter probe, like e.g., SYBR green based assays.

For interpretation of results, the exact state and nature of the DNA used must be considered e.g., recently killed cells (e.g., from disinfection) may still contain intact DNA, complicating interpretations in situations where quantifying viable bacteria carrying ARGs is the goal. Depending on the extraction method, even extracellular DNA can be detected. Damaged DNA that may not be capable of conferring or horizontally transferring gene function (e.g., due to fragmentation, strand breaks, etc.) can still be quantified given the short length of the amplification targets of the assays [29]. The limited calibration range of TaqMan qPCR or any other qPCR may also affect accurate quantification of very high or low ARG concentrations. The high cost and complexity of TaqMan probe-based assays can be prohibitive for large-scale studies and projects with limited research funds. Multiplexing could be one strategy to reduce costs by parallelizing analysis of several target genes. Several assays corresponding to gBlock1 (*mcr-1, aadA, aph(3″)-Ib, tetB*) and gBlock2 (*ermF, blaTEM, qnrS*) share the same optimized cycling conditions (annealing temperature of 60 °C and annealing time of 15 s). These assays therefore have the potential to be multiplexed, provided that probes with distinct fluorophores are designed, and the multiplexed application is tested and further optimized. In contrast, genes such as *mecA* and *ermF* could not be multiplexed under the optimized conditions established in this study.

## Related research article

None.

## For a published article

Not Applicable.

## Ethics statements

None.

## CRediT author statement

SR: Conceptualization, Primer and probe design, Methodology, Laboratory Experiments, Data collection, Formal Analysis, Writing – Original Draft. KB and DW: Primer and probe design, Data collection, Writing – Review & Editing. HB: Conceptualization, Supervision, Project Guidance, Funding acquisition, Writing – Review & Editing.

## Declaration of competing interest

The authors declare that they have no known competing financial interests or personal relationships that could have appeared to influence the work reported in this paper.

## Data Availability

Data will be made available on request.

## Appendix Supplementary materials

Supplementary material *and/or* additional information [OPTIONAL] Image, application 1
